# Protein Oxidation in Foods: Mechanisms, Consequences, and Antioxidant Solutions

**DOI:** 10.3390/foods10102346

**Published:** 2021-10-01

**Authors:** Mario Estévez, Youling L. Xiong

**Affiliations:** 1 IPROCAR Research Institute, University of Extremadura, 10003 Cáceres, Spain; 2 Department of Animal and Food Sciences, University of Kentucky, Lexington, KY 40546, USA

Protein oxidation in foods remains a topic of the utmost scientific interest. The first papers on this topic were written almost 30 years ago, when Wan et al. [1] reported how the protection of myofibrillar proteins against oxidation improved their functionality, and Martinaud et al. [2] described, for the first time, the occurrence of protein oxidation as a post-mortem event in aging beef. The awareness that protein oxidation could have a major impact on protein functionality [3] and beef quality [4] has led to the worldwide exploration of the chemical and biophysical mechanisms underlying the oxidative reaction occurring in muscle foods. While the research on this topic was initially concentrated on muscle foods, and some of the most cited review papers were published [3,5,6,7], other food scientists began to realize the ubiquity and similar oxidative changes occurring in non-muscle food systems. A retrospective assessment of the progress made in this research field reveals remarkable scientific breakthroughs, such as the manipulation of the redox properties of food proteins by using specific phytochemicals to improve the functionality of proteins [8] and the structural and rheological properties of muscle foods [9]. Furthermore, new studies have emerged to emphasize the potential threat to human health and mitigation strategies for dietary oxidized food proteins and amino acids [10].

The purpose of this Special Issue of *Foods*, entitled “Protein Oxidation in Foods: Mechanisms, Consequences, and Antioxidant Solutions”, was to compile some recent reports on protein oxidation that cover a broad range of food proteins from different agricultural commodities (muscle, dairy, plant, cereal, etc.) and diverse topics (chemistry, food processing, alleviation strategies, etc.). A historical approach is taken to provide the context of research in this rapidly evolving field.

In regard to the gain of further knowledge on the fundamentals of protein oxidation, Chen et al. [11] studied the molecular interactions of selected primary or secondary lipid oxidation products (LOP), namely linoleic acid 13-hydroperoxide (13-HPODE) and malondialdehyde (MDA), with whey protein isolate (WPI) stabilized in oil-in-water (O/W) emulsions. The authors reported precise chemical modifications in the WPI as a result of the interactions with oxidized lipids and concluded that the extent of oxidation and the subsequent structural changes depended on the formation of secondary LOP products and the protein position in the O/W emulsion systems. Théron et al. [12] contributed to the understanding of the chemistry and consequences of the oxidation of digestive enzymes by using synchrotron radiation circular dichroism (SRCD). The SRCD was found to be useful to study the rapid kinetics of protein folding and unfolding in real-time, giving highly resolved spectral data. A subsequent assessment of the proteolytic activity of oxidized pepsin was performed by MALDI-TOF mass spectrometry on a meat protein model.

Protein oxidation is known to be promoted during the handling, processing, and storage of foods, and several papers from this Special Issue look at the impact of some novel processing technologies. Sun et al. [13] studied the effectiveness of the application of ultrasonic freezing (UF) as a method to control protein oxidation, increase freezing speed, and improve the quality of frozen common carp (*Cyprinus carpio*). Air freezing (AF) and immersion freezing (IF) were used as controls in the study. The results showed UF inhibited protein oxidation (carbonyl and dityrosine content) caused by frozen storage, compared to AF and IF technologies. The authors concluded that UF was an efficient technique to mitigate the impact of protein oxidation on frozen fish muscle and preserve the gel properties of the proteins. Salting is a widespread technique of food preservation, but knowledge on the impact of NaCl on protein oxidative stability is limited. Hence, to provide further insight into the redox properties of NaCl on protein oxidation, Cao et al. [14] investigated the influence of increasing concentrations of NaCl (0–500 mM) on the oxidative stability of filled hydrogel stabilized with heat-denatured whey protein concentrate. Notably, higher NaCl concentrations significantly promoted the oxidation of lipids and proteins, hence affecting the oxidative stability of filled hydrogels. The results from this study could be used in future application for a optimized ionic strength conditions in emulsion-based foods. The combination of salting and air-drying is the technological basis for the production of numerous dry-cured meats. The occurrence of protein oxidation in dry-cured hams [15], loins [16], and sausages [17] have been previously described, yet, the paper from Bonifacie et al. [18] provides further insights into the interconnection between protein oxidation, nitrosation, and nitrosylation in dry-cured fermented sausages. The formation of nitroso compounds (NOCs), such as potentially mutagenic nitrosamines, nitrosylheme, and nitrosothiols, is a serious safety concern in cured muscle foods. The authors found that reducing the nitrite/nitrate levels from 0/200 or 120/120 to 80/80 in fermented sausages led to low lipid and protein oxidation levels and inhibited the formation of toxic NOCs. The application of high-voltage cold plasma has been proposed as a promising method for microbial inactivation at low temperatures, and hence it is used for decontamination of biological materials, including foods of an assorted nature [19]. The impact of such technology on protein oxidation is scarcely known. Olatunde et al. [20] studied the effects of in-bag dielectric barrier discharge high voltage cold plasma (IB-DBD-HVCP) on the oxidative stability of myofibrillar protein isolate (MPI) from Asian sea bass. The impact of MPI oxidation on its physiochemical and gelling properties were also addressed. According to their results, IB-DBD-HVCP treatment, particularly for 5 min, enhanced cross-linking of proteins in Asian sea bass myofibrillar proteins, resulting in improved gel elasticity and strength. 

Dairy proteins are known to be highly susceptible to oxidation, and the present Special Issue presents several noteworthy examples of innovative approaches to studying the oxidative impact on the functionality of this particular food protein group. UV-B illumination is commonly applied in the dairy industry as an effective means to prevent post-contamination after milk heat treatment, among other applications [21]. Photo-oxidation of food proteins is known to occur, yet, the impact of UV-B illumination on the oxidative stability and properties of dairy proteins is poorly understood. To provide new insights into this topic, Zhao et al. [22] examined the impact of UV exposure to oxidation-induced aggregation of apo-α-lactalbumin under aerobic and anaerobic conditions and the role of tryptophan (Trp) as a photosensitizer. The authors reported that the addition of Trp to apo-α-LA promoted the formation of covalently bound aggregates only under anaerobic conditions. The mechanistic insight obtained from this study could be useful for tailoring protein aggregates in future food applications. Working with WPI films, Wang and Xiong [23] contributed to the understanding of the underlying molecular interactions between selected phytochemicals (oxidized ferulic and tannic acids) and dairy proteins. Both oxidized acids induced protein oxidation and promoted WPI crosslinking through the actions of quinone carbonyl and protein sulfhydryl and amino groups. The incorporation of oxidized tannic acid, in particular, significantly reduced light transmittance and transparency of the WPI film and its in vitro digestibility. Moreover, plant proteins were coveredin this Special Issue by the paper presented by Xu et al. [24], who have studied Chiba tofu as a new vegetarian food produced from soy protein isolate (SPI). In particular, the authors assessed the effect of protein oxidation during storage on structural characteristics of SPI and rheology, texture, microstructure, and sensory properties of Chiba tofu. The onset of oxidation caused increases in carbonyl content and turbidity of SPI, while thiols were depleted over storage time. The oxidation modified SPI conformation, leading to a transition of α-helix and β-turn to β-sheet and random coil and the formation of protein crosslinks and aggregations. The time of storage had a remarkable impact on the extent and consequences of protein oxidation. Considering the results from the sensory evaluation, Chiba tofu stored for 12 days had the highest overall quality score.

The Special Issue also includes studies aimed to protect muscle foods against protein oxidation by means of natural antioxidants. The growing interest of food scientists in phenolic-rich extracts is obvious and a recent comprehensive review provides critical insights into the application of these natural antioxidants as bioactive ingredients in meat and meat products [25]. Morcuende et al. [26] applied sprayable extracts from selected fruits, namely, oaknut (*Quercus ilex* subsp. ballota), rose hips (*Rosa canina* L.), common hawthorn (*Crataegus monogyna* Jacq.), and strawberry tree (*Arbutus unedo* L.) to lamb cutlets to counteract the negative impact of protein oxidation that occurred during cold storage in a high oxygen modified atmosphere packaging. While the four fruits showed relevant antioxidant potential, common hawthorn had the highest phenolics and tocopherol content, and that was reflected in efficient antiradical activity. Taking the results altogether, this fruit was also found to be most efficient in protecting lamb cutlets from lipid oxidation. In regard to protein oxidation, oaknut was the most efficient in protecting lamb cutlets against protein carbonylation as a plausible involvement of ellagitannins. Spraying lamb cutlets with extracts from oaknut, rose hips, and common hawthorn improved consumers’ purchase intention for chill stored lamb cutlets. On the other hand, de Santana Neto et al. [27] focused on a relatively unknown fruit (yellow mombin (*Spondias mombin* L.)) as a source of antioxidant ingredients in ready-to-eat chicken patties. The extract from the the fruit was found effective in maintaining red color and inhibiting myoglobin degradation in the evaluated samples. Furthermore, the treated samples had significantly lower amount of lipid and protein oxidation products as compared to the control counterparts. Therefore, the phenolic-rich extracts from yellow mombin bagasse could be used as a potential natural preservative and extend shelf-life of ultraprocessed muscle foods.

The Special Issue is crowned with a review paper in which Xiong and Guo [28] provide an overview of the theory, evolution, and recent developments in protein oxidation research across different food commodity groups. The paper is outlined with an updated revision of protein oxidation mechanisms and means of detection, in which the most recent discoveries of the fundamental chemistry and biochemistry of oxidative reactions are unveiled. The authors provide a comprehensive update of the impact of protein oxidation on the functionality of diverse food proteins and address the management of industrial processes to improve the technological properties of oxidatively modified proteins. Finally, the authors collect latest information on antioxidant strategies applied in situ to both animal and and plant food production.

In conclusion, this Special Issue of *Foods*, entitled “Protein Oxidation in Foods: Mechanisms, Consequences and Antioxidant Solutions”, compiles relevant and updated research works and illustrates the current state of knowledge of the subject. The volume includes a broad field of topics, from molecular fundamentals and mechanisms to applicative studies aiming to understand the technological impact of food protein oxidation and suggesting means to control and manage this ubiquitous oxidative phenomenon in food processing and product quality optimization.
